# Directed Evolution of Protein-Based Sensors for Anaerobic Biological Activation of Methane

**DOI:** 10.3390/bios14070325

**Published:** 2024-06-30

**Authors:** Ehsan Bahrami Moghadam, Nam Nguyen, Yixi Wang, Patrick C. Cirino

**Affiliations:** Department of Chemical and Biomolecular Engineering, University of Houston, Houston, TX 77204-4004, USA; ebahrami@cougarnet.uh.edu (E.B.M.);

**Keywords:** alkylsuccinate synthase, methylsuccinate, itaconate, ItcR

## Abstract

Microbial alkane degradation pathways provide biological routes for converting these hydrocarbons into higher-value products. We recently reported the functional expression of a methyl-alkylsuccinate synthase (Mas) system in *Escherichia coli*, allowing for the heterologous anaerobic activation of short-chain alkanes. However, the enzymatic activation of methane via natural or engineered alkylsuccinate synthases has yet to be reported. To address this, we employed high-throughput screening to engineer the itaconate (IA)-responsive regulatory protein ItcR (WT-ItcR) from *Yersinia pseudotuberculosis* to instead respond to methylsuccinate (MS, the product of methane addition to fumarate), resulting in genetically encoded biosensors for MS. Here, we describe ItcR variants that, when regulating fluorescent protein expression in *E. coli*, show increased sensitivity, improved overall response, and enhanced specificity toward exogenously added MS relative to the wild-type repressor. Structural modeling and analysis of the ItcR ligand binding pocket provide insights into the altered molecular recognition. In addition to serving as biosensors for screening alkylsuccinate synthases capable of methane activation, MS-responsive ItcR variants also establish a framework for the directed evolution of other molecular reporters, targeting longer-chain alkylsuccinate products or other succinate derivatives.

## 1. Introduction

Methane and short-chain alkanes are abundant feedstocks in the chemical and energy industries. The controlled activation and conversion of these hydrocarbons into value-added products remains a major technical hurdle [1,2]. Microbial alkane degradation pathways provide biological routes to activate and metabolically convert small gaseous alkanes into higher-value products [3,4,5], offering a potential solution to the large-scale, capital-intensive challenges associated with existing gas-to-liquid technologies (e.g., Fischer-Tropsch) [1]. Anaerobic bio-activation of *n*-alkanes occurs predominantly through their addition to the double bond of fumarate via the activity of alkylsuccinate synthase enzymes [6]. The alkylsuccinate products are further degraded through rearrangement, decarboxylation, and β-oxidation, coupled with fumarate regeneration [7,8]. A key challenge to taking advantage of these pathways lies in the difficulty of functionally expressing alkylsuccinate synthases, along with their partner “activating” enzymes (AEs), in a host organism suitable for metabolic engineering and bioprocessing. We recently demonstrated the first-ever functional expression of methyl-alkylsuccinate synthase (Mas) from the *Azoarcus* sp. strain “HxN1”, along with its partner AE, in a recombinant host (*Escherichia coli*) [4]. Consistent with the Mas activity reported for HxN1 [6], we observed the activation of C3-C6 linear alkanes.

Organisms capable of anaerobic degradation of ethane and propane have been identified [5,8,9], but there are no indications of alkylsuccinate synthase-based methane activation in nature. Having established the functional expression of Mas, we now seek to improve the biosynthesis of alkylsuccinates as well as engineer Mas for (enhanced) activity toward shorter alkanes and, potentially, methane. Given the many variables and possible bottlenecks in this transformation, it is desirable to employ a high-throughput screening of many different gene libraries to identify mutations that confer enhanced product formation. High-throughput screening is also a powerful approach for identifying enzyme variants having improved substrate specificity, or even activity toward non-native substrates. In this context, a common limitation in high-throughput mutational analysis is the lack of a sensitive and compound-specific screening method. For the case of alkylsuccinates produced within a mixture of many dicarboxylic acids, we know of no such assay. The use of natural or engineered bacterial transcription factors as endogenous biosensors that report on the production of target small molecules is now an established, powerful approach to screening for novel or improved biosynthesis in whole cells [10,11,12]. Here, we describe the design of genetically encoded biosensors that specifically report on the presence of methylsuccinate (MS, the product of methane addition to fumarate) via the expression of a fluorescent reporter protein.

**Figure 1:**
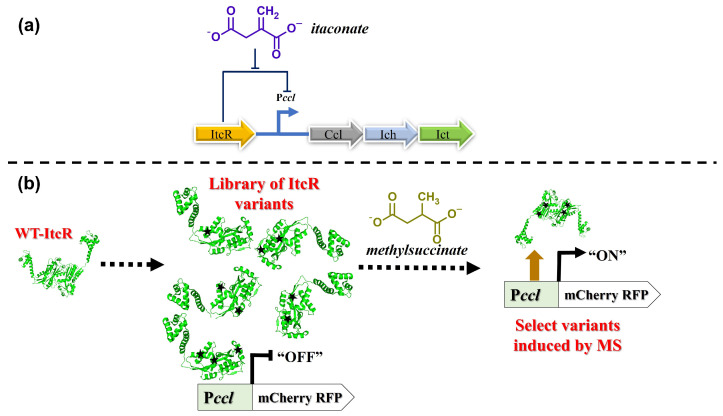
Regulatory mechanisms of ItcR and variant selection strategy. (**a**) Transcriptional regulation by ItcR from *Y. pseudotuberculosis*: ItcR represses the transcription of genes under the control of the promoter P*ccl*. Repression is relieved upon binding itaconate (IA). *Ccl*, *Ich*, and *Ict* are *Y. pseudotuberculosis* genes involved in itaconate catabolism [13]. (**b**) ItcR variants showing an enhanced induced response to methylsuccinate (MS) were isolated by placing a reporter gene (that encodes the mCherry red fluorescent protein) under the control of P*ccl*, enabling the high-throughput screening of variants libraries.

These biosensors were constructed by altering the ligand specificity of the ItcR repressor. ItcR is a LysR-type transcriptional regulator located in the opposite direction of an operon encoding genes responsible for the catabolism of itaconate (IA, also called methylenesuccinate(2-)) in *Yersinia pseudotuberculosis* [13]. Gene repression by ItcR is relieved when the repressor binds itaconate [13]. Itaconate differs from MS by just one C-C bond (Figure 1), yet ItcR shows a 100-fold lower induced gene expression response to MS at 5 mM inducer [13]. We tested whether wild-type ItcR (WT-ItcR), when expressed in *E. coli*, could be induced by other alkylsuccinates of interest: (1-ethyl)succinate (ES), (1-methylethyl)succinate (MES), and (1-methylpentyl)succinate (MPS). In all cases, the response was lower than that observed for MS (Table S2). Given the chemical similarity of itaconate to MS, we reasoned that ItcR could be engineered to instead respond to MS without great difficulty. Indeed, a single round of random mutagenesis yielded an ItcR variant that, when expressed in *E. coli*, shows a >9-fold improved response to 1 mM MS (added to the culture broth). Subsequent rounds of mutagenesis resulted in a collection of other variants having further enhanced sensitivity and specificity toward MS. The structural modeling and analysis of the ItcR ligand binding pocket provides mechanistic insights into the altered molecular recognition. In addition to serving as a tool to screen for the Mas-variant-catalyzed, oxygen-independent activation of methane, MS-responsive ItcR variants also serve as starting points for further directed evolution of inducer specificity toward other alkylsuccinates (products of fumarate addition to short-chain alkanes) and succinate derivatives.

## 2. Materials and Methods

### 2.1. Materials and Reagents

Itaconic acid (purity ≥ 99.0%) and methylsuccinic acid (purity ≥ 99.0%) were procured from TCI America (Portland, OR, USA) and utilized in the screening assays. ES was acquired from Enamine (Kyiv, Ukraine). MES and MPS were synthesized as previously described [4]. T4 DNA ligase and all restriction enzymes were obtained from New England Biolabs (Ipswich, MA, USA), with T4 ligase specifically employed in ligation reactions. NEBuilder^®^ HiFi DNA Assembly Master Mix, also sourced from New England Biolabs, was used for Gibson Assembly [14]. The high-fidelity PCR reactions in this study were conducted using either Phusion^®^ High-Fidelity DNA Polymerase or Q5^®^ High-Fidelity DNA Polymerase, supplied by New England Biolabs. PCR conditions were determined based on the NEB Tm Calculator (https://tmcalculator.neb.com/) and adhered to the manufacturer’s instructions for the respective polymerases. The GeneMorph II Random Mutagenesis Kit, obtained from Agilent Technologies (Santa Clara, CA, USA), was employed for the random mutagenesis of the target gene. For gel purification of DNA fragments, Zymoclean™ Gel DNA Recovery Kit from Zymo Research (Irvine, CA, USA) was utilized.

### 2.2. Strains and Plasmids

All experimental procedures, including cloning, fluorescence-activated cell sorting (FACS), plate screening, and specificity characterization, were performed using the MC1061 *E. coli* strain. In this study, the mCherry fluorescent protein, a widely used red fluorescent protein (RFP), was utilized due to its stability, brightness, and minimal toxicity to cells [15]. Green fluorescent protein (GFP) has been used previously, where its high expression levels resulted in reduced cell density, confirming the reported toxicity of elevated GFP expression in *E. coli* cells [16]. In our hands, no such problem was observed with mCherry. The use of this reporter protein in conjunction with transcription factor-based sensors is well documented to provide high sensitivity and specificity in detecting target metabolites [17,18,19]. Plasmid pPCC2102 (Ptac-ItcR, P*ccl*-GFP) was constructed by replacing AraC-TAL and PBAD from pPCC1322 (Ptac-AraC-TAL, PBAD-GFP) [20] with the genes encoding wild-type ItcR and the P*ccl* promoter (from *Y. pseudotuberculosis*, accession number: CP032566.1) [13]. Plasmid pPCC2106 (Ptac-ItcR) was constructed by removing the GFP gene. Plasmid pPCC2107 (Ptac-ItcR, Pccl-RFP) (Figure S1) was constructed by replacing the GFP gene with the mCherry (RFP) gene sequence [15] (accession number: AY678264.1).

### 2.3. Library Construction

The gene encoding ItcR’s ligand-binding domain (LBD) was subjected to random mutagenesis via error-prone PCR (pPCC2107 used as the template) utilizing the Agilent GeneMorph II Random Mutagenesis Kit, resulting in random (RM) library 1. The most sensitive variant (Var1) from RM library 1 was subsequently used as the parent to construct RM library 2. The error rates for the libraries were determined by sequencing ten clones selected randomly from each library. These rates for RM libraries 1 and 2 were approximately 2.75 mutations/kb and 4.2 mutations/kb, respectively. The mutations that appeared in Var1 were removed individually via a Gibson assembly [14] of multiple PCR fragments to construct Var2, Var3, and Var4. Also, the combinatorial assembly of mutations identified in Var5 to 7 was performed by assembling purified PCR fragments amplified from each variant, resulting in the construction of Var8 and Var9. A similar strategy to random mutagenesis (RM) with slight variations was applied to generate two site-saturation mutagenesis (SSM) libraries. For SSM1, Var1 was amplified using oligonucleotides containing degenerated codons (NDT) in the locations of the two beneficial mutations (127AA and 144AA). Then, for SSM2, Var8 was amplified using the same approach but targeting the 173AA and 280AA locations. The number of possible variants from each of these SSM libraries is 144.

### 2.4. Fluorescence-Activated Cell Sorting (FACS)

A volume of 3 mL of LB medium with an antibiotic (Apr) was inoculated with 30 μL of glycerol stock and cultivated (6 h, 37 °C, 250 rpm). This starter culture was diluted in 3 mL of fresh LB medium with an antibiotic (to reach OD_600_~0.1) and incubated for 1 h. Then, the culture was induced by adding the inducer (MS) to reach a final concentration of 1 mM. The culture was then incubated for 15 h at 37 °C with continuous shaking at 250 rpm. Prior to sorting, each culture was diluted five-fold with filtered LB medium. Fluorescence characteristics were determined, and cell sorting was performed using a FACS AriaII cell sorter with a 70 µm nozzle size and a sheath pressure of 70 psi in the Cytometry and Cell Sorting Core facility at Baylor College of Medicine. To detect mCherry fluorescence, 600 nm long-pass and 610/20 nm band-pass filters were used during the sorting process. The excitation and emission wavelengths applied in the sorting were 567 nm and 600 nm, respectively. Cells were identified by gating in the SSC-A (side-scatter characteristics) vs. FSC-A (forward-scatter characteristics) plot. Doublets were excluded from the analysis by gating in the FSC-H vs. FSC-A and SSC-H vs. SSCW plots. Dead cells were eliminated by gating in SSC-A vs. DAPI (4′,6-diamidino-2-phenylindole). In the first round, the lower 30.2% of the least fluorescent cells were selected during the negative screening (without MS). This population was then used to run the positive screening (with MS), which resulted in sorting of the upper 0.016% of the most fluorescent cells (Figure S2). For the second round, a similar approach was employed, with 79.3% of the least fluorescent cells and 0.43% of the most fluorescent cells isolated in the negative and positive screening, respectively. Cells were collected into 10 mL of LB medium containing an antibiotic, centrifuged for 30 min at 4 °C and 4000 rpm, and resuspended in 4 mL of SOC for 1 h at 37 °C and 250 rpm. The cells were then grown overnight and stored as glycerol stocks. FlowJo™ v10.8 Software [21] was used to analyze sorting data.

### 2.5. Rescreening and Characterization of Isolated Variants

After each round of FACS, the regenerated sorted cells were grown on agar plates containing an antibiotic. Individual colonies were then inoculated into 500 µL of LB medium with an antibiotic in 96-deep-square-well plates (MASTERBLOCK^®^, 96 WELL, 2 ML, PP, V-BOTTOM, Greiner Bio-One (Kremsmünster, Austria)) and incubated at 37 °C and 900 rpm for 6 h. Afterward, 20 μL of each culture was transferred into 480 μL of LB in two different plates: one without the inducer and one with 1 mM inducer. These plates were incubated at 37 °C and 900 rpm for 15 h. These cultures were then centrifuged, and the cell pellets were resuspended in 500 μL of PBS. This process (centrifugation and resuspension) was repeated. The resulting cell suspensions were diluted in PBS (4-fold) before measuring OD_600_ and fluorescence intensity (excitation and emission wavelengths set to 587 nm and 610 nm, respectively). Variants representing an improved response compared to the parent were isolated and re-cloned into a fresh RFP (mCherry) vector. The re-cloned variants underwent an additional RFP fluorescence assay to validate their initial improved response. This screening process is illustrated in Figure S3.

Dose–response and inducer specificity were determined using 96-well-plate cultures and fluorescence plate reader assays, essentially as described for variant rescreening. Normalized fluorescence values reported from 96-well plate measurements represent the measured fluorescence (RFU) divided by the cell density (OD_600_). Fold induced RFP fluorescence values indicate the normalized fluorescence in the presence of the inducer, divided by the normalized fluorescence value in the absence of the inducer.

### 2.6. Structural Modeling and Molecular Docking

The crystal structure of the LBD of ItcR has been determined, both with and without the binding of itaconate [22]. Additionally, we generated structural models of both WT-ItcR and Var7 using AlphaFold (v2.3) [23]. Maestro 13.4 [24] was used to perform molecular docking, employing the Glide module [25]. Both the S-MS and R-MS isomers were docked into the predicted LBD structure of Var7. Protein structures were prepared using the Protein Preparation Wizard in Maestro, which involved adding hydrogen atoms, assigning partial charges, and optimizing the hydrogen-bonding network. Water molecules beyond 5 Å from hetero groups were deleted, and the protein structure was minimized using the OPLS4 force field until the average root-mean-square deviation (RMSD) of the non-hydrogen atoms reached 0.3 Å. Ligands were prepared using the LigPrep module in Maestro, which involved generating ionization states at a physiological pH of 7.0 ± 2.0, considering possible conformers, and performing energy minimization using the OPLS4 force field. The receptor grid was generated using the Receptor Grid Generation tool in Glide, with the grid box centered on the LBD of ItcR. The default inner box size of 10 Å and outer box size of 20 Å were used. Docking simulations were carried out using the standard precision (SP) mode in Glide, with the van der Waals scaling factor for the receptor and ligand set to 0.8 and ligand sampling set to flexible. A maximum of 20 poses per ligand were generated, and poses were ranked based on the GlideScore. As a control, IA was docked into the LBD of the WT-ItcR structure (IA-bound state, PDB: 7W07). The docked position of IA is nearly identical to that from the solved structure (RMSD of 0.58 Å, based on carbon atoms, Figure S7). Docking poses that fell within the score range of the control (Table S4) were deemed to be feasible final binding modes of MS-Var 7 (LBD). The 3D structures of both IA and MS were acquired from the PubChem database [26]. Also, all structural visualizations of protein–ligand complexes in this study were generated using PyMOL v2.0 [27].

## 3. Results and Discussion

### 3.1. Directed Evolution of MS-Responsive ItcR Variants

**Figure 2:**
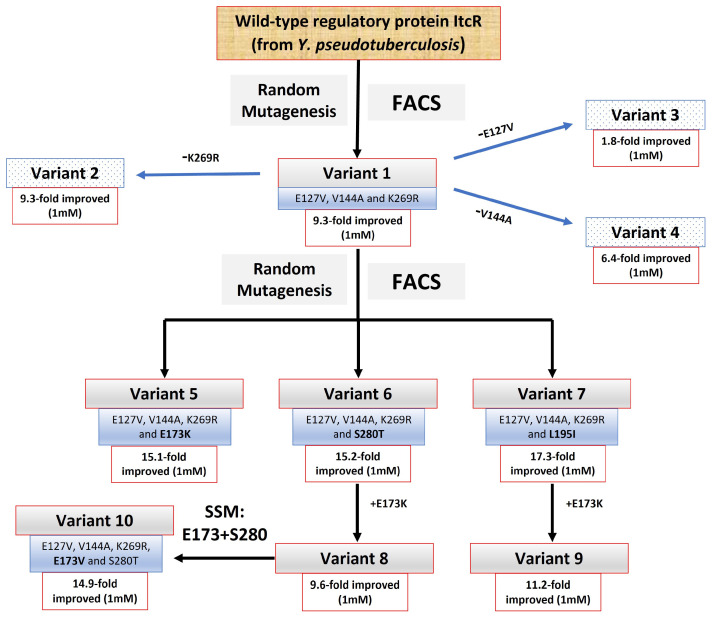
Phylogenetic tree showing the evolution of MS-responsive variants originating from WT-ItcR. The indicated fold improved values represent the fold enhancement in mCherry RFP fluorescence in the presence of the indicated concentration of MS relative to WT-ItcR. SSM: site-saturation mutagenesis.

The evolution progression of MS-sensitive variants originating from WT-ItcR is depicted in Figure 2. We initially used random mutagenesis across the entire ItcR ligand-binding domain (LBD) coding region. Screening was performed using FACS, with the induced expression of mCherry red fluorescent protein (RFP) [15] serving as the reporter. To remove “leaky” variants that cause high RFP fluorescence in the absence of the inducer, random mutagenesis libraries were first sorted to isolate a low-background population in the absence of MS. This population was next subjected to “positive” screening in the presence of 1 mM MS. First-round screening resulted in variant #1 (“Var1”) (E127V, V144A, and K269R), showing 9.3 ± 0.9-fold improvement in transcriptional response to 1 mM MS. We next checked the contribution of each individual amino acid substitution found in Var1 toward this improved response by constructing variants #2, #3, and #4 (“Var2”, etc.). Var2 (E127V and V144A) showed the same response as Var1, while Var3 (V144A and K269R) and Var4 (E127V and K269R) showed a 5.2 ± 0.8-fold and 1.5 ± 0.2-fold lower induced gene expression response to 1 mM MS (relative to Var1), respectively. This result led us to generate a site-saturation mutagenesis (SSM) library targeting AA positions 127 and 144, using Var1 as the parent. Here, “NDT” codons were used, resulting in a library consisting of 144 possible variants, out of which 400 isolated clones were screened. No variant showed an improved response relative to Var1.

A second round of random mutagenesis (with Var1 as parent) and FACS sorting (using 1 mM MS) was next conducted. The resulting variants, Var5 (E127V, V144A, K269R, and E173K), Var6 (E127V, V144A, K269R, and S280T), and Var7 (E127V, V144A, K269R, and L195I), showed 15.1 ± 2-, 15.2 ± 0.9-, and 17.3 ± 2-fold improvement in the RFP expression response to 1 mM MS (relative to WT-ItcR), respectively. From these newly identified amino acid substitutions, we next constructed variants Var8 (E127V, V144A, K269R, S280T, and E173K) and Var9 (E127V, V144A, K269R, L195I, and E173K). The E173K substitution was added to Var6 and Var7 because Var5 showed the highest response to a relatively low concentration of MS (100 µM). Although Var8 and Var9 showed lower-fold induced RFP at the high concentration (1 mM), Var8 showed a 1.2 ± 0.2-fold improved response to 100 µM MS compared to Var5. This result, in turn, guided the generation of another SSM library, this time targeting positions 173 and 280, with Var8 as the parent. The library was screened in the presence of 1mM MS, yielding Var10 (E127V, V144A, K269R, and E173V) showing 1.6 ± 0.3-fold induced RFP expression in 1 mM MS (as compared to Var8), but no improvement with 100 µM MS.

### 3.2. Characterization of MS Biosensors

Dose–response curves were fitted to a modified Hill equation [28], as shown below:

(1) $$y=a+\left(b-a\right)/(1+{\left(\frac{k}{x}\right)}^{n})$$

**Figure 3:**
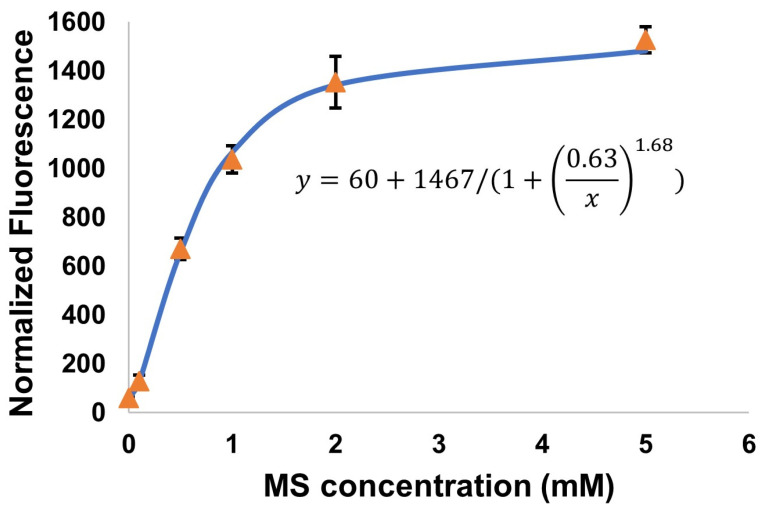
MS-induced RFP expression in *E. coli* harboring the “Var7” MS biosensor system. Normalized fluorescence intensity (“y”, RFU/OD_600_) is plotted against MS concentration (that which was added to the culture broth), “x”. Data were fitted to Equation (1), as shown (R^2^ > 0.99).

Here, *y* is the response (i.e., the normalized fluorescence of cells, resulting from the expression of RFP) at *x* concentration of the inducer (note that this is the concentration of the *exogenous* inducer—that which was added to the culture broth). Parameters *a*, *b*, and *k* describe the response at zero concentration, the maximum response, and the concentration corresponding to 50% of the maximum response (may be considered an “apparent K_d_” for the response to the exogenous inducer), respectively. The Hill coefficient (*n*) describes the cooperativity of the biosensor. The calculated values of these parameters for each designed biosensor are reported in Table 1. The corresponding plotted data and fitted equations are provided in Figure S6 of the Supplementary Materials. One representative dose–response curve (that for Var7) is provided in Figure 3.

WT-ItcR, and presumably its variants, operates as a dimer with two possible ligand binding sites. We determined a Hill coefficient of ~1.7 for WT-ItcR with IA (Table 1). Most variants similarly have Hill coefficients in this range (~1.5–1.9) with MS, suggesting a conserved cooperative binding mechanism. The values for Var8 and Var9 are lower but still indicative of positive, partial cooperativity. Such variation in *n* may reflect alterations in the binding site and/or protein conformation that affect ligand binding cooperativity.

**Figure 4:**
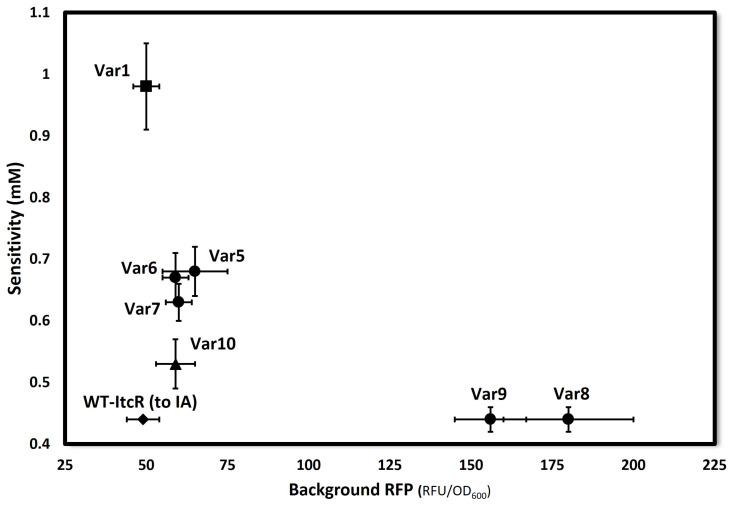
Sensitivity (mM) vs. background RFP (RFU/OD_600_) of each ItcR variant. Sensitivity is defined as the concentration at half of the saturation signal (‘*k*’ in Equation (1)). Background refers to the absolute normalized fluorescence measured in the absence of the inducer. Data are the average of 3 values, and error bars represent the range. ♦ represents WT-ItcR, with IA as the inducer; ■ represents variant Var1; ● represents responsive variants derived from Var1; ▲ represents Var10, derived from SSM.

The sensitivity of each variant sensor system, quantified by ‘*k*’ in Equation (1), is plotted against their respective background RFP expression levels (parameter ‘*a*’, representing “leaky” expression) in Figure 4. All ItcR variants show enhanced sensitivity to MS. Var8 and Var9 display sensitivities to MS comparable to that of WT-ItcR to IA (~0.44 mM), though notably with much higher leaky expression. All other variants show background levels comparable to that of WT-ItcR.

Table 2 lists the fold induced RFP expression value for each variant at 0.1 mM, 1 mM, and 5 mM MS. Var7 shows a ~25-fold induced expression response to 5 mM MS, which represents a 10-fold improvement compared to WT-ItcR at the same concentration.

The inducer specificity of the MS-responsive ItcR variants was assessed by comparing the RFP expression response to MS with that to IA, as well as other, potentially “competing” ligands when screening for MS biosynthesis (i.e., fumarate and succinate). As shown in Table 3, all biosensors show high specificity toward MS over both fumarate and succinate (≥12). Specificity over IA is also significantly improved compared to WT-ItcR, with Var6 having ~77-fold increased MS/IA specificity.

### 3.3. Binding-Site Modeling and Analysis: Variant 7 vs. WT-ItcR

The structures of the WT-ItcR and Var7 LBDs were modeled using AlphaFold [23]; this was performed prior to the publication of the solved ItcR X-ray crystal structure [22]. The predicted structure exhibited remarkable similarity to the experimentally solved structure, with an RMSD of 0.64 Å. The predicted LBD structure of Var7 (using AlphaFold) deviated from the WT-ItcR LBD structure by an RMSD of 0.66 Å, suggesting the amino acid substitutions in Var7 did not likely confer substantial structural changes to the LBD. IA and MS (both R- and S-isomers) were next docked into the LBDs of WT-ItcR and Var7 (refer to Methods Section). The resulting Glide docking parameters are provided in Table S4. Detailed docking poses for WT-ItcR with IA, WT-ItcR with MS, Var7 with IA, and Var7 with MS (R- and S-isomers), including relevant interaction distances, are provided in Figure S8.

**Figure 5:**
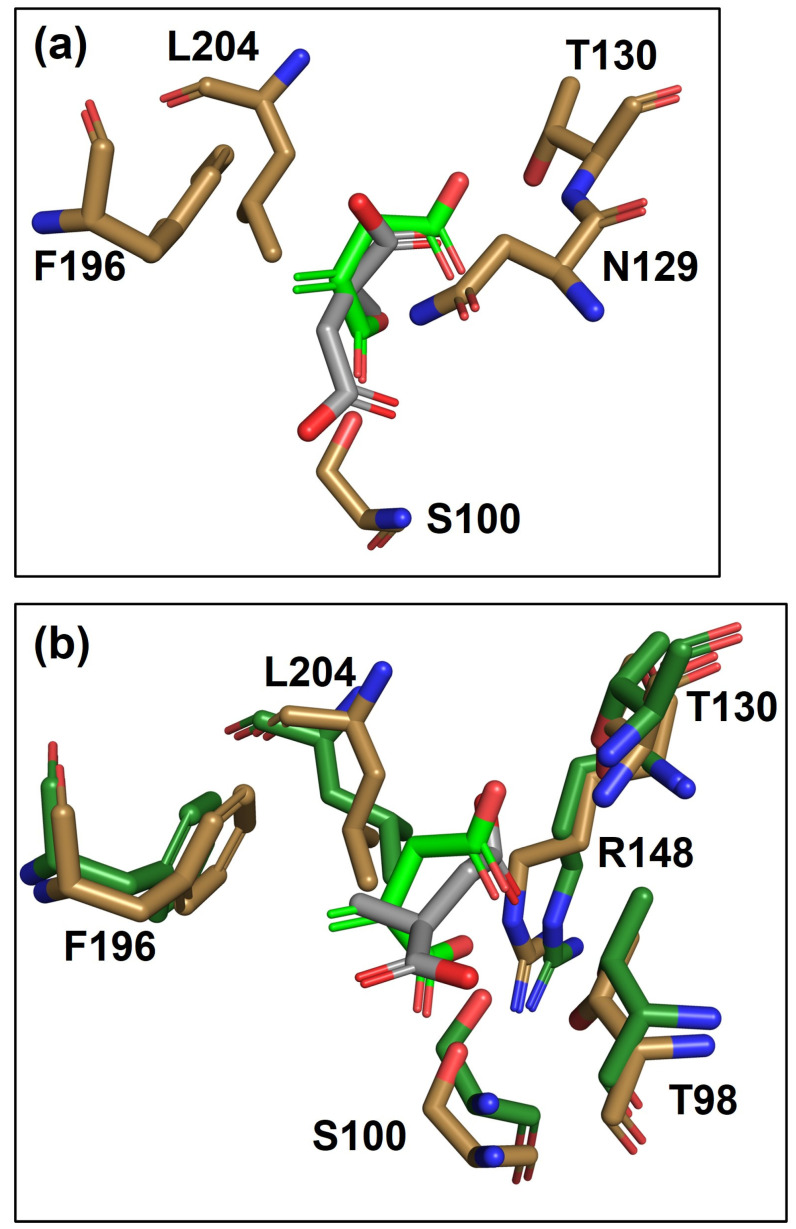
Overlays of binding pocket residues and ligand poses resulting from the molecular docking of (**a**) WT-ItcR with MS (S-isomer) vs. IA, and (**b**) Var7 with MS (S-isomer) vs. WT-ItcR with IA. WT-ItcR and Var7 binding pocket residues are shown in sand and dark green, respectively. C-C bonds in IA and MS are depicted in light green and gray, respectively. O atoms are red, and N atoms are blue. For ease of visualization, only the most relevant binding pocket residues are included. More detailed structures are presented in Figure S8.

Figure 5 depicts docking pose overlays for WT-ItcR with IA vs. MS (S-isomer) and WT-ItcR with IA vs. Var7 with MS. For ease of visualization, only the most relevant binding pocket residues are included in these overlays. In comparing the docking pose of IA to that of MS in WT-ItcR (Figure 5a), there is a clear difference in ligand orientation. Perhaps most notably, whereas the methylene group of IA lies 3.9 Å above the F196 ring (forming a critical hydrophobic interaction [22]), the methyl group of MS is instead pointing away from F196. The Glide docking scores (Table S4) indicate stronger binding for IA in WT-ItcR (−5.32 kcal/mol) as compared to MS (−4.01 kcal/mol). It is important to note that while MS is a much poorer inducer of WT-ItcR as compared to IA, MS still acts as a binding pocket ligand. 

Figure 5b shows a clear shift in the position of MS in Var7 relative to that in WT-ItcR. For reference, the C-atom RMSD between IA and MS docked into WT-ItcR is 2.97 Å, while that between IA in WT-ItcR and MS in Var7 is 1.62 Å. Significantly, we now see the MS methyl group positioned 4.2 Å above F196 in the Var7-MS complex, similar to the methylene of IA in the WT structure. The L195I substitution of Var7 likely helps to reposition F196 to support this interaction. L195I may further alleviate steric hindrance by the slightly bulkier MS ligand. It is also noteworthy that the T98 hydroxyl group in Var7 now lies 2.8 Å from O3- of MS (the same distance from O2- of IA in the WT-ItcR complex), as compared to 4.1 Å between these atoms for the case of MS docked into WT-ItcR. H-bonding interactions with R148 and S100 are similarly more conserved between the Var7-MS and WT-ItcR-IA complexes as compared with those for MS docked into WT-ItcR.

## 4. Conclusions

The efficient conversion of methane to liquid fuels and other value-added chemicals remains the “holy grail” of catalysis; the conversion of short-chain alkanes is similarly challenging. Enzymatic/microbial approaches hold promise in overcoming the many catalytic hurdles and inefficiencies associated with existing chemical process technologies [29]. Whereas oxygen-dependent biological processes suffer from inherently large carbon and energy losses, anaerobic methane/alkane activation and conversion may significantly improve efficiency [1]. Toward the directed evolution of enzyme-based methane activation via fumarate addition, here, we report the first-ever genetically encoded biosensors of MS based on variants of the ItcR repressor. The differential responsivity of WT-ItcR to IA (high) and MS (negligible) compared to the Var7 sensor’s response—high to MS and reduced to IA—highlights the ability of subtle structural changes resulting from relatively few amino acid substitutions to significantly impact ligand binding and allosteric regulation. In addition to serving as a tool that enables high-throughput screening of mutant gene libraries for novel MS biosynthesis, an ItcR-based MS sensor serves as a “parent” in the further directed evolution of biosensors for alkylsuccinates resulting from the addition of fumarate to ethane and other short-chain alkanes.

## Figures and Tables

**Table 1:** Dose–response parameters of ItcR variants (with inducer MS) and WT-ItcR (with inducer IA), resulting from data fitted to Equation (1) (R^2^ > 0.99).

Variant	*a*	*b*	*k* (mM)	*n*
Var1	50 ± 4	870 ± 50	0.98 ± 0.10	1.9 ± 0.1
Var5	65 ± 10	1500 ± 100	0.68 ± 0.04	1.5 ± 0.1
Var6	59 ± 4	1350 ± 20	0.67 ± 0.04	1.7 ± 0.1
Var7	60 ± 4	1500 ± 50	0.63 ± 0.03	1.7 ± 0.1
Var8	180 ± 20	2300 ± 90	0.44 ± 0.02	1.3 ± 0.1
Var9	160 ± 10	2300 ± 70	0.44 ± 0.02	1.4 ± 0.1
Var10	59 ± 6	1200 ± 40	0.53 ± 0.04	1.7 ± 0.3
WT-ItcR (to IA)	49 ± 5	2700 ± 20	0.44 ± 0.01	1.7 ± 0.1

**Table 2:** Fold induced RFP expression response to 0.1, 1, and 5 mM MS, for indicated ItcR variants ^1^.

MS Concentration (mM)	WT	Var1	Var5	Var6	Var7	Var8	Var9	Var10
0.1	1.0 ± 0.1	1.2 ± 0.2	2.2 ± 0.4	1.8 ± 0.2	2.2 ± 0.6	2.7 ± 0.1	2.4 ± 0.2	1.9 ± 0.2
1	1.0 ± 0.1	9.3 ± 0.9	15 ± 2	15 ± 1	17 ± 2	9.6 ± 0.9	11 ± 1	15 ± 2
5	2.5 ± 0.2	17 ± 1	23 ± 3	23 ± 1	25 ± 3	13 ± 1	14 ± 1	20 ± 2

^1^ Fold induced RFP indicates the normalized fluorescence value in the presence of the inducer divided by the normalized fluorescence value in the absence of the inducer. Data are the average of 3 values ± SD.

**Table 3:** Inducer specificity of ItcR variants ^1^.

	WT	Var1	Var5	Var6	Var7	Var8	Var9	Var10
MS/IA	0.030 ± 0.002	1.4 ± 0.1	1.0 ± 0.1	2.3 ± 0.1	1.4 ± 0.1	1.8 ± 0.1	0.8 ± 0.1	1.1 ± 0.1
MS/fumarate	1.8 ± 0.1	15 ± 1	23 ± 4	21 ± 4	23 ± 3	12 ± 2	12 ± 1	15 ± 2
MS/succinate	1.9 ± 0.3	17 ± 2	22 ± 3	25 ± 2	27 ± 6	13 ± 3	13 ± 1	16 ± 1

^1^ Specificity is defined as the ratio of fold increased RFP expression in 2 mM MS over fold increased RFP in the same concentration of the alternate compound (IA, fumarate, or succinate). Data are the average of 3 values ± SD.

## Data Availability

The data presented in this study are available on request from the corresponding author.

## Supplementary Materials

The following supporting information can be downloaded at https://www.mdpi.com/article/10.3390/bios14070325/s1: Figure S1: Plasmid map of pPCC2107 (reporter plasmid); Figure S2: Sorting histograms for first round of screening using FACS; Figure S3: Sequential screening protocol for selection of ItcR mutants; Figure S4: Change in fold induction vs. MS concentration for variants; Figure S5: Fold induced RFP vs. MS concentration for variants; Figure S6: MS-induced RFP expression in E. coli harboring the MS biosensor system; Figure S7: An overlay of the IA molecule, which was obtained through docking, with its experimentally determined location; Figure S8: Structural analysis of ItcR and variant Var7 with corresponding ligands; Table S1: Primers used in this study; Table S2: Fold induced RFP vs. alkylsuccinate concentration for WT-ItcR; Table S3: Fold induced RFP vs. MS concentration for variants; Table S4: The Glide docking result parameters. References [15,22] are cited in the Supplementary Materials.
