# Supplementary material for: Directed Evolution of Protein-Based Sensors for Anaerobic Biological Activation of Methane
Source: Biosensors (Basel). 2024 Jun 30;14(7):325. doi: 10.3390/bios14070325 (PMC11275114; doi:10.3390/bios14070325)
Supplement: Supplementary file 1 [file biosensors-14-00325-s001.zip › biosensors-3050741-supplementary.pdf]

Supplementary Materials

Table of Contents

Figure S1. Plasmid map of pPCC2107 (reporter plasmid) **2**

Full sequence of pPCC2107 (reporter plasmid)..**2**

Table S1. Primers used in this study **4**

Figure S2. Sorting histograms for the first round of screening using FACS........................**5**

Figure S3. A comprehensive diagram of the sequential screening protocol **5**

Figure S4. Change in fold induction vs. MS concentration for each variant **6**

Figure S5. Fold induced RFP vs. MS concentration for each variant...................................**6**

Figure S6. MS-induced RFP expression in *E. coli* harboring the MS biosensor system ………………**7**

Figure S7. A comparative overlay of itaconate (IA) positioning obtained via docking vs. its experimentally determined location **8**

Figure S8. Structural analysis of ItcR and variant Var7 with corresponding ligands........**9**

Table S2. Fold induced RFP vs. alkylsuccinates concentration (mM) for WT-ItcR..........**11**

Table S3. Fold induced RFP vs. MS concentration (mM) for each variant **11**

Table S4. The Glide docking result parameters. **12**

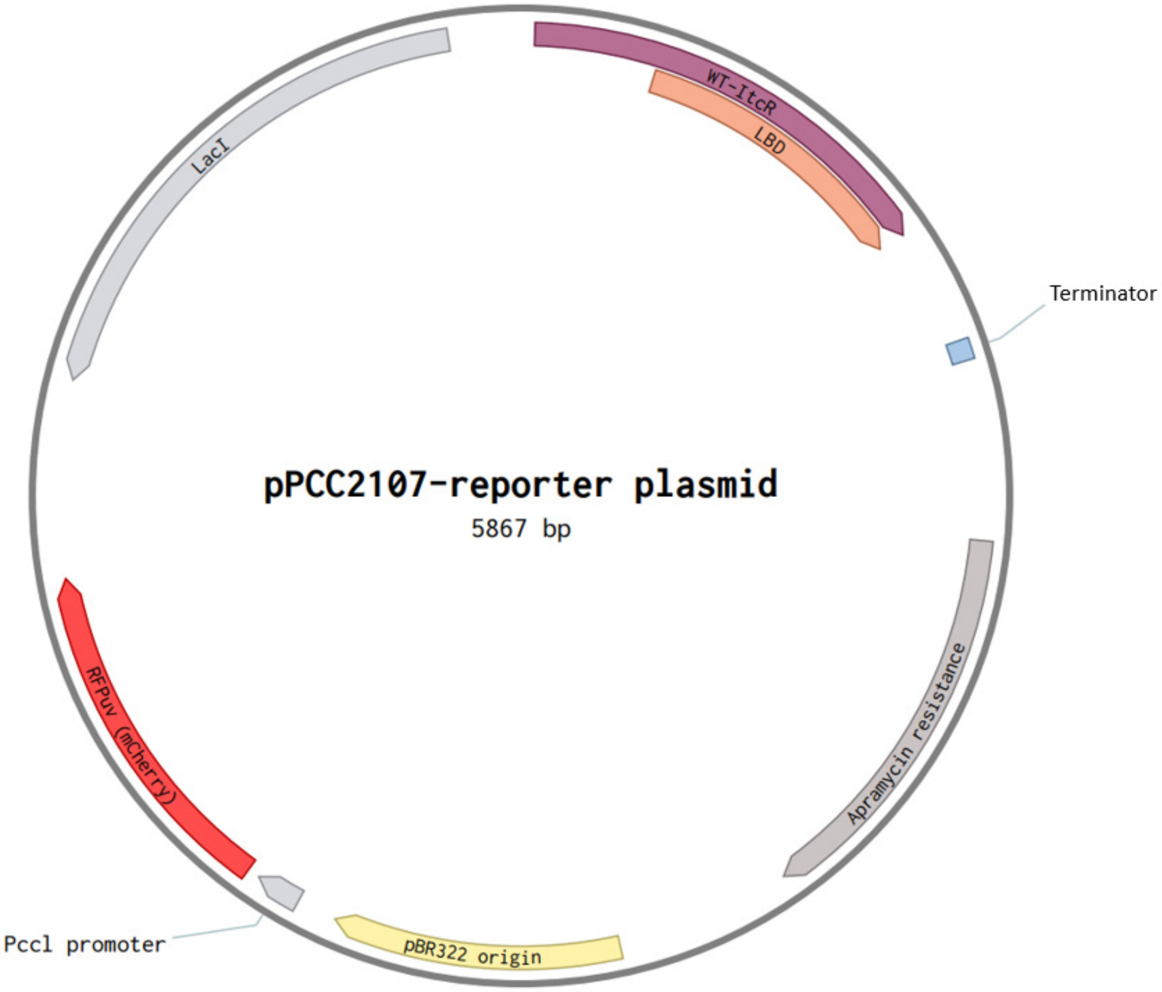

Figure S1. Plasmid map of pPCC2107 (reporter plasmid).

Full sequence of pPCC2107 (reporter plasmid): Sequences encoding the DNA-binding domain (DBD) and the ligand-binding domain (LBD) of the ItcR regulatory protein [1] are shown in green and purple, respectively. Additionally, the coding sequence for the mCherry protein [2] is indicated in red:

taacaatttcacacaggagatacctaggatggagttacgtcatatacgctattttctggctgttgctgaagaacgacatttcacacgcgctgccgcccggctaggaatagggcagcctcctctcagtc
agcaaattaaggatctggagagggaactcggtgcgttgttgttccggcgggtttcttatggcgcagaattaactgaggcaggtattgcatttctggatgttgtcaaagagatacctgtgatggcaga
aagagcgacccaagctgcgcagcgggctgtcaggggagaactcggcgtactcagagttggctttactgcgtcatcagcatttaacagcgtagtgcctacggcgatccgtgcctttagacgtgcat
atcctgatgtacgattacagctagaggaagataacacgactcgactggctgatggattgaatgaaggctcgctcgatgtcgctttcctgaggccaggctttgctggcagcgaacgtttccacctcag
aatgctatctgaagagccgatgatgattgtcatggcggaaaatcacccggcggcatcgtatgaagaaatttcgcttagcgcattcagggatgaaactttcctgctatttccacgagagattggtctga
cgctttacgattcagtgattgagtcatgccgtactgcgggttttgagccgactattggccaactggcaccacaaatcgcttcggtcattaatctcgttgcggcagaaatgggcgtttcaatcgttccgg
cttccatgagccaagtgaaagttattggggttgtatatcgacacattgctgaccagacgccgacagcaaagctcgcgttggcgtaccggcgaggggatacctcgccggtattaagaaactttgtcc
tgaccgtgtttccttgattggtaacgaatcagacaattgacggcttgacggagtagcatagggtttgcagaatccctgcggtaccagatctgtcgactacaaggacgatgacgacaagtgaagatc
gatctctcgatcgagtgagagaagacttgcatgcctgcaggtcgactctagaggatccccgtactatcaacaggttgaactgcggatcttgcggccagctttatgcttgtaaaccgttttgtgaaaaa
atttttaaaataaaaaaggggacctctagggtccccaattaattagtaatataatctattaaaggtcattcaaaaggtcatccaccggatcaattcccctgctcgcgcaggctgggtgccaagctctcg
ggtaacatcaaggcccgatccttggagcccttgccctcccgcacgatgatcgtgccgtgatcgaaatccagatccttgacccgcagttgcaaaccctcactgatccggctcacggtaactgatgccg
tatttgcagtaccagcgtacggcccacagaatgatgtcacgctgaaaatgccggcctttgaatgggttcatgtgcagctccatcagcaaaaggggatgataagtttatcaccaccgactatttgcaa
cagtgccgttgatcgtgctatgatcgactgatgtcatcagcggtggagtgcaatgtcgtgcaatacgaatggcgaaaagccgagctcatcggtcagcttctcaaccttggggttaccccggcggt
gtgctgctggtccacagctccttccgtagcgtccggcccctcgaagatgggccacttggactgatcgaggccctgcgtgctgcgctgggtccgggagggacgctcgtcatgccctcgtggtcaggt
ctggacgacgagccgttcgatcctgccacgtcgcccgttacaccggaccttggagttgtctctgacacattctggcgcctgccaaatgtaaagcgcagcgcccatccatttgcctttgcggcagcgg
ggccacaggcagagcagatcatctctgatccattgcccctgccacctcactcgcctgcaagcccggtcgcccgtgtccatgaactcgatgggcaggtacttctcctcggcgtgggacacgatgcca
acacgacgctgcatcttgccgagttgatggcaaaggttccctatggggtgccgagacactgcaccattcttcaggatggcaagttggtacgcgtcgattatctcgagaatgaccactgctgtgagc
gctttgccttggcggacaggtggctcaaggagaagagccttcagaaggaaggtccagtcggtcatgcctttgctcggttgatccgctcccgcgacattgtggcgacagccctgggtcaactgggc
cgagatccgttgatcttcctgcatccgccagagggcgggatgcgaagaatgcgatgccgctcgccagtcgattggctgagctcatgagcggagaacgagatgacgttggagggggcaaggtcg
cgctgattgctggggcaacacatcattgcagcactggggccagatggtaagccctcccgtatcgtagttatctacacgacggggagtcaggcaactatggatgaacgaaatagacagatcgctga
gataggtgcctcactgattaagcattggtaactgtcagaccaagtttactcatatatactttagattgatttaaaacttcatttttaatttaaaaggatctaggtgaagatcctttttgataatctcatgacca
aaatcccttaacgtgagttttcgttccactgagcgtcagaccccgtagaaaagatcaaaggatcttcttgagatcctttttttctgcgcgtaatctgctgcttgcaaacaaaaaaaccaccgctaccagc
ggtggtttgtttgccggatcaagagctaccaactctttttccgaaggtaactggcttcagcagagcgcagataccaaatactgtccttctagtgtagccgtagttaggccaccacttcaagaactctgt
agcaccgcctacatacctcgctctgctaatcctgttaccagtggctgctgccagtggcgataagtcgtgtcttaccgggttggactcaagacgatagttaccggataaggcgcagcggtcgggctg
aacggggggttcgtgcacacagcccagcttggagcgaacgacctacaccgaactgagatacctacagcgtgagctatgagaaagcgccacgcttcccgaagggagaaaggcggacaggtatc
cggtaagcggcagggtcggaacaggagagcgcacgagggagcttccagggggaaacgcctggtatctttatagtcctgtcgggtttcgccacctctgacttgagcgtcgatttttgtgatgctcgt
caggggggcggagcctatggaaaaacgccagcaacgcggcctttttacggttcctggccttttgctggccttttgctcaacttttcatactcccgccattcagagcttcatatccaaaagcaattaaac
acaccggtatcatatattggataaatgataacggcgaccatagactaagcgaagttggaggaggaaccatacccgggagttgcaggataatcaaaatggtgagcaagggcgaggaggataac
atggccatcatcaaggagttcatgcgcttcaaggtgcacatggagggctccgtgaacggccacgagttcgagatcgagggcgagggcgagggccgcccctacgagggcacccagaccgcca
agctgaaggtgaccaagggtggcccccctgcccttcgcctgggacatcctgtcccctcagttcatgtacggctccaaggcctacgtgaagcaccccgccgacatccccgactacttgaagctgtcctt
ccccgagggcttcaagtgggagcgcgtgatgaacttcgaggacggcggcgtggtgaccgtgacccaggactcctccctgcaggacggcgagttcatctacaaggtgaagctgcgcggcacca
acttcccctccgacggccccgtaatgcagaagaagaccatgggctgggaggcctcctccgagcggatgtaccccgaggacggcgccctgaagggcgagatcaagcagaggctgaagctgaa
ggacggcggccactacgacgctgaggtcaagaccacctacaaggccaagaagcccgtgcagctgcccggcgcctacaacatcaacatcaagttggacatcacctcccacaacgaggactacac
catcgtggaacagtacgaacgcgccgagggccgccactccaccggcggcatggacgagctgtacaagtaaggggggatccactagttctagagcggccgccaccgcggtggagctccagctttt
gttccctttagtgagggttaattgcgcaaacacagaaaaaagcccgcacctgacagtgcgggcttttttttttgcaactgttgggaagggcgatcggtgcgggcctcttcgctattacgccagctggc
gaaaggggggatgtgctgcaaggcgattaagttgggtaacgccagggttttcccagtcacgacgttgtaaaacgacggccagtgaatccgtaatcatggtcatagctgtttcctgtgtgaaattgtta
tccgctcacaattccacacaacatacgagccggaagcataaagtgtaaagcctggggtgcctaatgagtgagctaacttacattaattgcgttgcgctcactgcccgctttccagtcgggaaacctgt
cgtgccagctgcattaatgaatcggccaacgcgcggggagaggcggtttgcgtattgggcgccagggtggtttttcttttcaccagtgagacgggcaacagctgattgcccttcaccgcctggccc
tgagagagttgcagcaagcggtccacgctggtttgccccagcaggcgaaaatcctgtttgatggtggttaacggcgggatataacatgagctgtcttcggtatcgtcgtatcccactaccgagatat
ccgcaccaacgcgcagcccggactcggtaatggcgcgcattgcgcccagcgccatctgatcgttggcaaccagcatcgcagtgggaacgatgccctcattcagcatttgcatggtttgttgaaaac
cggacatggcactccagtcgccttcccgttccgctatcggctgaatttgattgcgagtgagatatttatgccagccagccagacgcagacgcgccgagacagaacttaatgggcccgctaacagcg
cgatttgctggtgacccaatgcgaccagatgctccacgcccagtcgcgtaccgtcttcatgggagaaaataatactgttgatgggtgtctggtcagagacatcaagaaataacgccggaacattag
tgcaggcagcttccacagcaatggcatcctggtcatccagcggatagttaatgatcagcccactgacgcgttgcgcgagaagattgtgcaccgccgctttacaggcttcgacgccgcttcgttctac
catcgacaccaccacgctggcacccagttgatcggcgcgagatttaatcgccgcgacaatttgcgacggcgcgtgcagggccagactggaggtggcaacgccaatcagcaacgactgtttgccc
gccagttgttgtgccacgcggttgggaatgtaattcagctccgccatcgccgcttccactttttcccgcgttttcgcagaaacgtggctggcctggttcaccacgcgggaaacggtctgataagagac
accggcatactctgcgacatcgtataacgttactggtttcacattcaccaccctgaattgactctcttccgggcgctatcatgccataccgcgaaaggttttgcaccattccatgggaattcgctagcgc
ggccgcgagctgttgacaattaatcatcggctcgtataatgtgtggaattgtgagcgga

Table S1. Primers used in this study.

Primer name	Sequence (5′ to 3′)
Upper BAD promoter-fw1	tctgctaatcctgttaccag
Upper BAD promoter-rv1	ctctgaatggcgggagta
ItcR-geneblock-fw1	ggataacaatttcacacagg
ItcR-geneblock-rv1	tctagagtcgacctgcag
Initial-Pccl fw	tactcccgccattcagag
Initial-Pccl rv	tagcccaaaaaaacgggtat
EP-ItcR-LBD-fw1	gcgcagcgggctgtc
EP-ItcR-LBD-rv1	cgtcaattgtctgattcgttaccaatca
Flank-ItcR-LBD-fw1	tgattggtaacgaatcagacaattgacg
Flank-ItcR-LBD-rv	gacagcccgctgcgcagctt
Upper BAD promoter(overhang)-rv1	ctctagaactagtgctctgaatggcgggagta
Mcherry(RFP)-fw2	acccgggagttgcaggataatcaaaatggtgagcaagggcgaggaggataa
Mcherry(RFP)-rv1	ctagaactagtggatccccttacttgtacagctcgtccatgcc
Pccl-Mcherry-rv1(overhang)	atcctgcaactcccgggtatggttcctcctccaacttc
2107-17var-Nhei-fw1	aaaggttttgcaccattccatgggaattcgctag
2107-17var-Xmni-rv1	tccccttttgctgatggagctgcacatgaacc
2107-17var-V144A-rv1	cctcaggaaagcgacatcgagcgagccttcattcaatccatcag
2107-17var-E127V-rv1	cagtcgagtcgtgttatcttcctctagctgtaatcgtacatcaggatatgcacgtc
2107-17var-K269R-fw1	cgacagcaaagctcgcgttggcgtaccggcgaggggatacct

2107-17var-E127V-fw1	agctagaggaagataacacgactcgactggctgatggattga
2107-51-1b-L195I-rv1	ctcgtggaaatatcaggaaagtttcatccctgaatgcgctaagcg
2107-51-1b-S280T-rv1	gcgtggtatcccctcgccggtacgcc
2107-51-1b-S280T-fw1	ggcgaggggataccacgccggtattaagaaactttgtcctgaccgtg
2107-51-1b-L195I-fw1	aactttcctgatatttccacgagagattggtctgacgctttacgat
2107-17-SSM1-rv1	agcgagccttcattcaatccatcagccagtcgagtcgtgttatcAHNctctagctgtaatcgtacatcaggatatgcacgtctaaaggcacggat
2107-17-SSM1-fw1	tggattgaatgaaggctcgctcgatNDTgctttcctgaggccaggctttgctggcag
2107-17-SSM2-rv1 (E173K)	cgccatgacaatcatcatcggctcttc
2107-17-SSM2-fw1 (E173K)	gccgatgatgattgtcatggcgNDTaatcacccggcggcatcgtatgaaga
2107-17-SSM2-rv2 (S280T)	ggtatcccctcgccggtacgcc
2107-17-SSM2-fw2 (S280T)	ggcgtaccggcgaggggataccNDTccggtattaagaaactttgtcctgaccgtgt

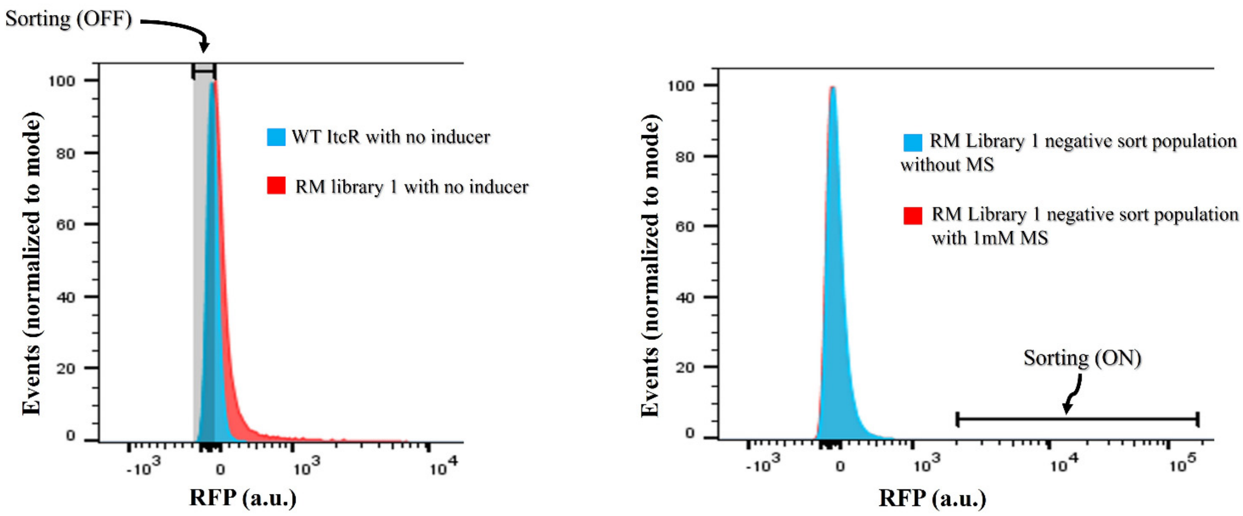

Figure S2. Sorting histograms for the first round of screening using FACS: 30.2% of the least fluorescent cells in the absence of MS were retained (left). Of these, 0.016% of the most fluorescent cells in the presence of 1 mM MS were isolated (right).

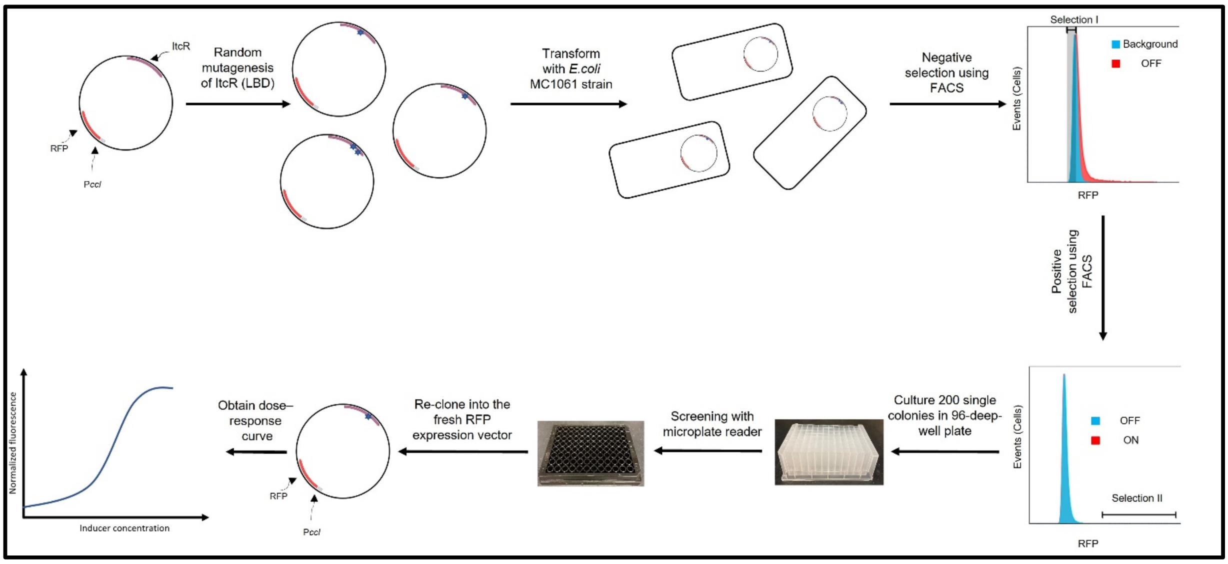

Figure S3. A comprehensive diagram of the sequential screening protocol for the selection of ItcR mutants, encompassing stages from mutagenesis to fluorescence-based sorting and sensitivity characterization.

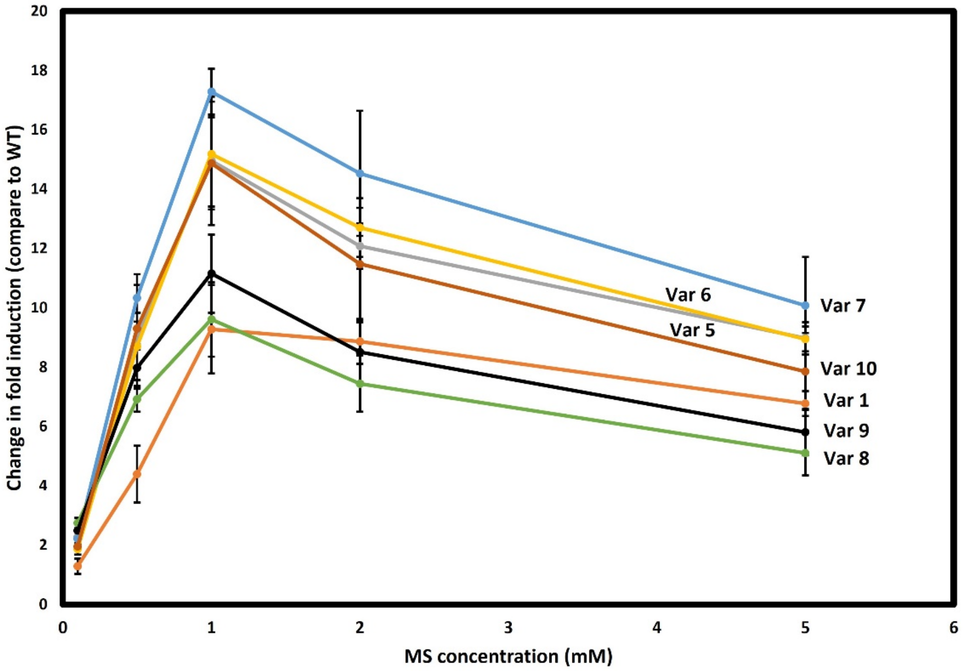

Figure S4. Change in fold induction vs. MS concentration for each variant. Change in fold induction is defined as the fold induced RFP of each variant divided by fold induced RFP of WT-ltcR. Data points are the average of 3 values, and error bars represent the range.

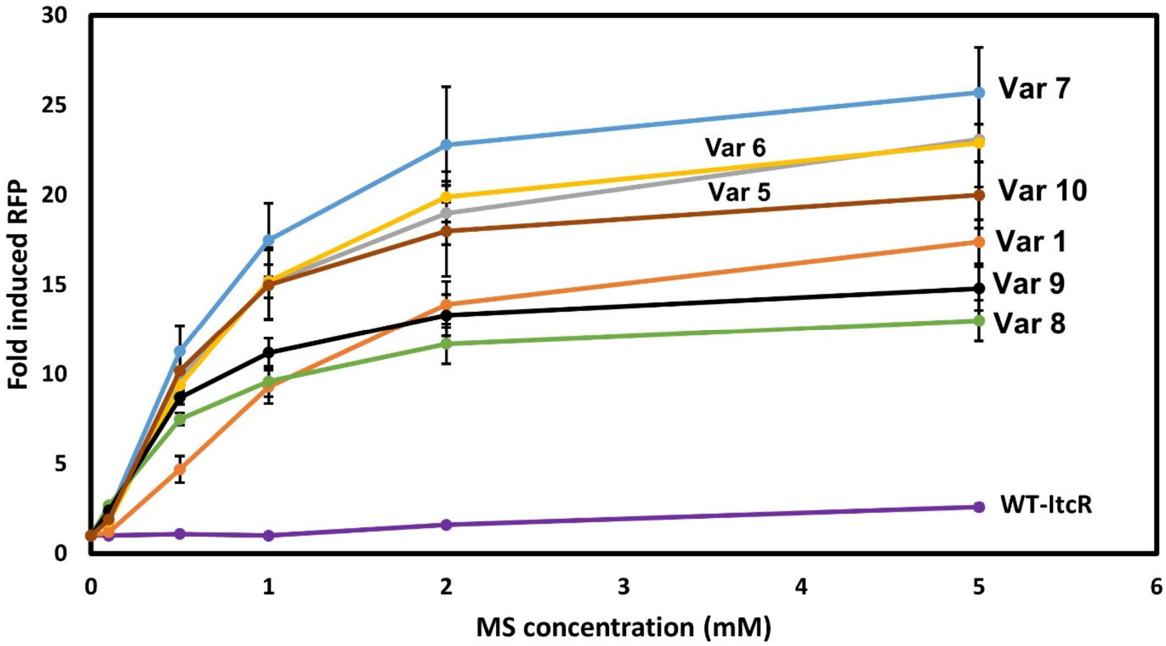

Figure S5. Fold induced RFP vs. MS concentration for each variant. Fold induced RFP indicates the normalized fluorescence value in the presence of the inducer divided by the normalized fluorescence value in the absence of the inducer. Data points are the average of 3 values, and error bars represent the range.

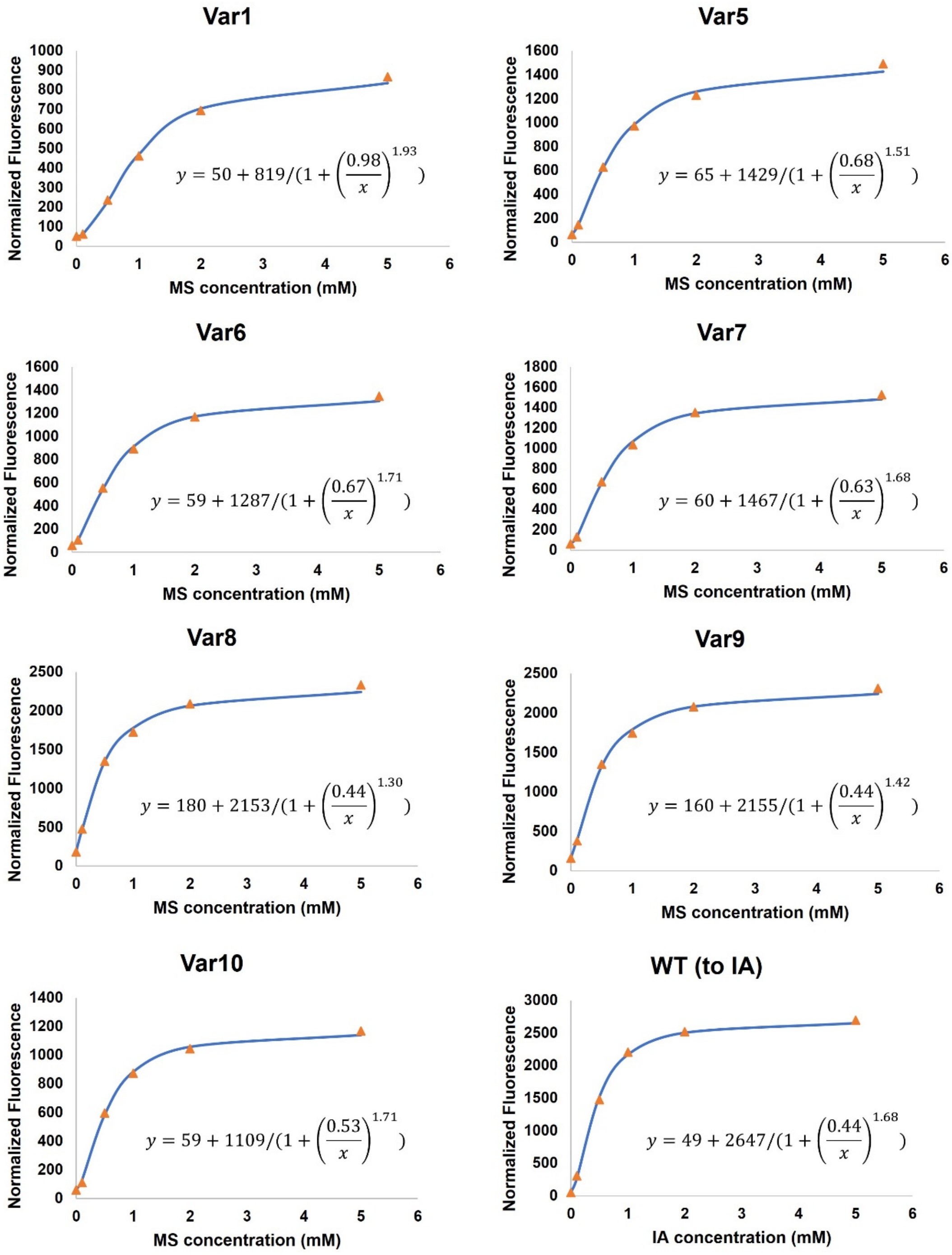

Figure S6. MS-induced RFP expression in E. coli harboring the MS biosensor system. Normalized fluorescence intensity ("y", RFU/OD600) is plotted against MS concentration (that which was added to the culture broth), "x". Data were fitted to Equation 1, as shown (R2>0.99). The response of WT-ItcR to IA is also shown.

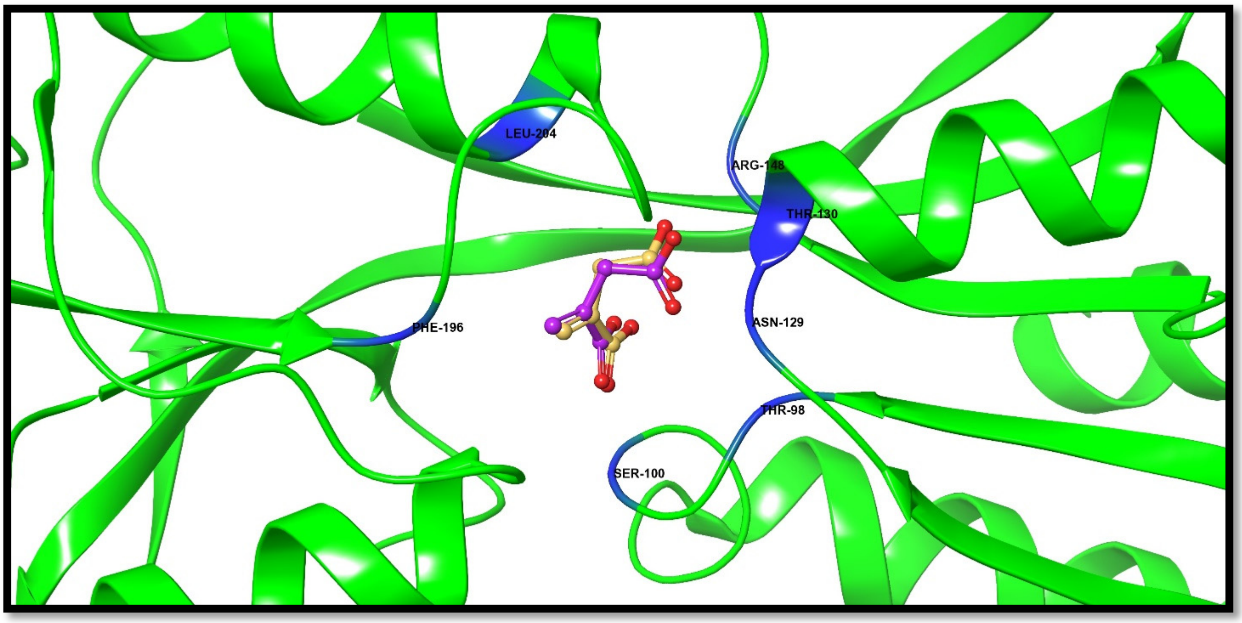

Figure S7. A comparative overlay of itaconate (IA) positioning obtained via docking vs. its experimentally determined location in the ligand-binding domain (LBD) of wild-type ItcR [1]. The blue residues represent IA binding sites within the LBD of WT-ItcR. The plum-colored itaconate illustrates the molecule's position as obtained through computational docking, while the yellow itaconate denotes the experimentally reported position of the molecule within the ItcR structure [1].

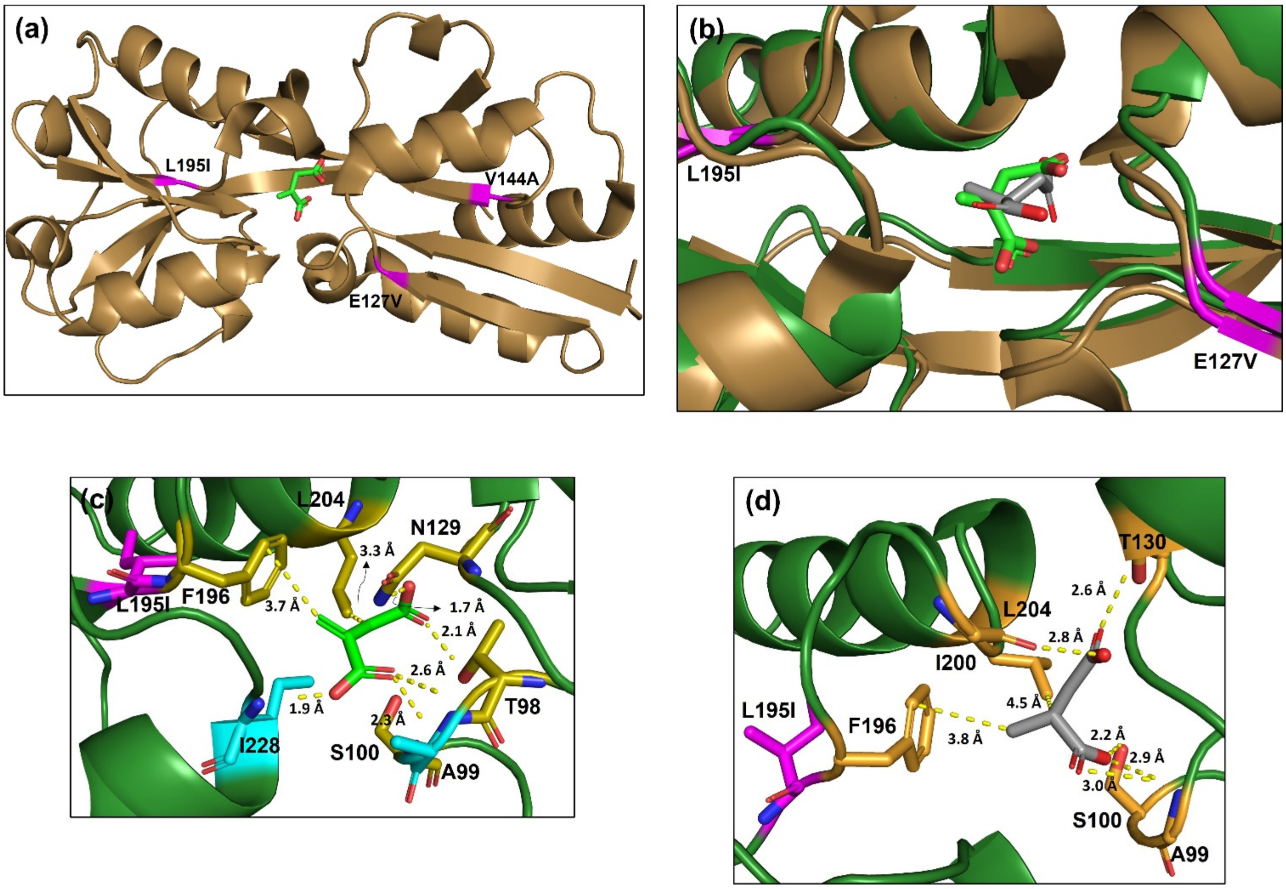

(a)
L195I
V144A
E127V
(b)
L195I
E127V
(c)
L204
N129
3.3 Å
L195I
F196
3.7 Å
1.7 Å
2.1 Å
2.6 Å
1.9 Å
I228
2.3 Å
T98
S100
A99
(d)
T130
2.6 Å
L204
2.8 Å
I200
4.5 Å
L195I
F196
3.8 Å
2.2 Å
2.9 Å
3.0 Å
S100
A99

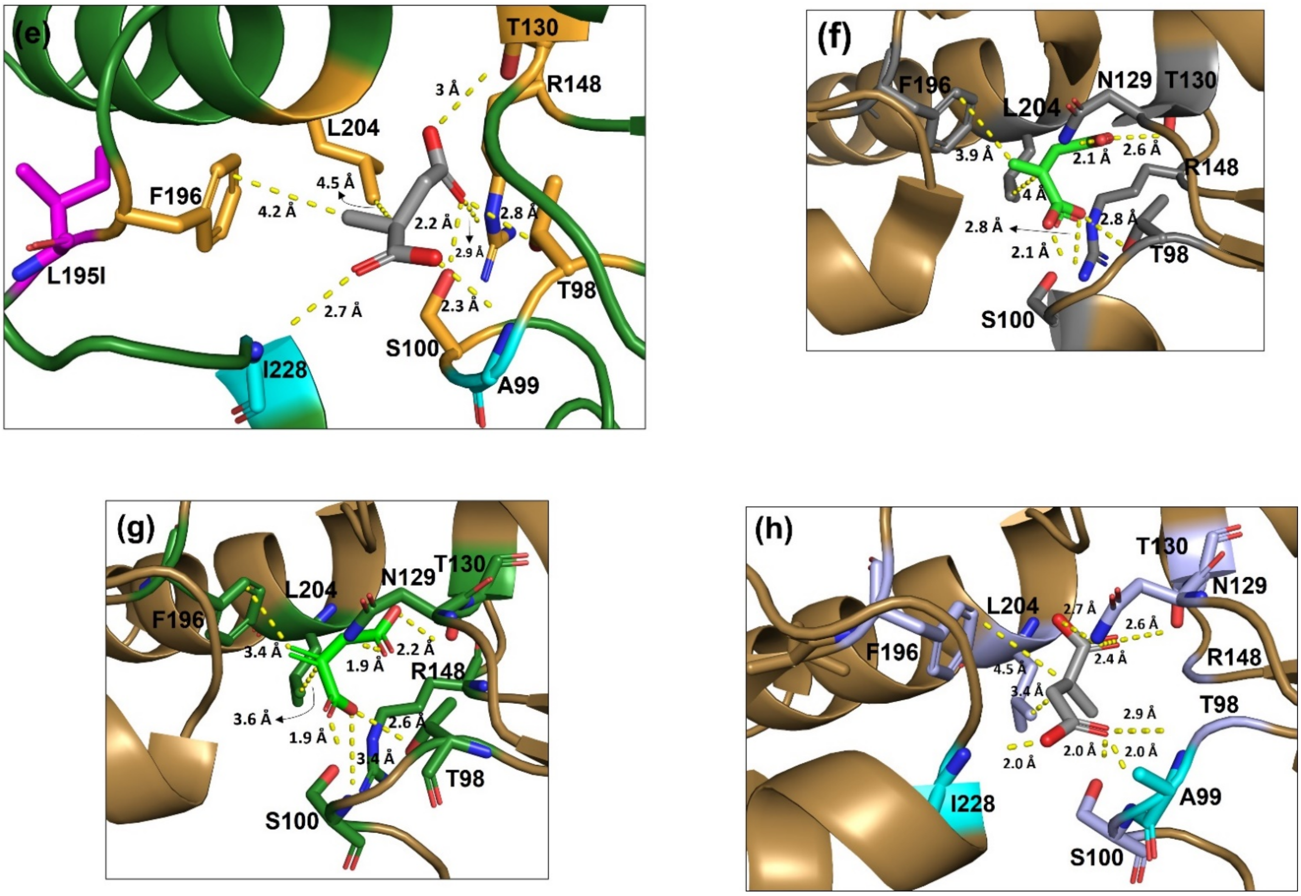

Figure S8. Structural analysis of ItcR and variant Var7 with the corresponding ligands. WT-ItcR is represented in sand yellow, and Var7 in forest green. C-C bonds in IA and MS are depicted in light green and gray, respectively. Non-neutral AA substitutions in Var7 are depicted in magenta. Yellow dashed lines indicate distances between atoms. (a) The structure of WT-ItcR LBD with IA as the ligand. (b) A comparison of binding sites between the WT-ItcR-IA complex [1] and the Var7-MS (S-isomer) complex. (c) The docking pose of IA within the Var7 LBD. Residues interacting with IA that are conserved in the IA binding of WT-ItcR (solved structure) are highlighted in olive. Non-conserved residues unique to IA-Var7 interactions and distinct from the IA binding of WT-ItcR residues are depicted in cyan. (d) The docking pose of the MS R-isomer within the Var7 LBD. Residues interacting with MS that are conserved in IA binding (solved structure) are highlighted in bright orange. (e) The docking pose of the MS S-isomer within the Var7 LBD. Residues interacting with MS that are conserved in IA binding (solved structure) are highlighted in bright orange. Non-conserved residues unique to MS interactions and distinct from IA binding residues are depicted in cyan. (f) The IA binding site in the WT-ItcR LBD structure [1]. Residues interacting with IA are accentuated in gray. (g) The docking pose of the IA within the WT-ItcR LBD. Residues interacting with IA that are conserved in IA binding (solved structure) are accentuated in dark green. (h) The docking pose of the MS S-isomer within the WT-ItcR LBD. Residues interacting with MS that are conserved in IA binding (solved structure) are highlighted in light blue. Non-conserved residues unique to MS interactions and distinct from IA-binding residues are depicted in cyan.

Table S2. Fold induced RFP vs. alkylsuccinate concentration (mM) for WT-ItcR. Fold induced RFP indicates the normalized fluorescence value in the presence of the inducer divided by the normalized fluorescence value in the absence of the inducer. Data points are the average of 3 values.

Concentration (mM)	Fold induced RFP of the WT-ItcR toward alkylsuccinates of interest (N=3, ±SD)		
	Alkylsuccinates		
	ES	MES	MPS
0	1.00±0.0	1.00±0.0	1.00±0.0
0.1	1.00±0.1	1.12±0.1	0.94±0.1
0.5	1.12±0.1	0.97±0.1	0.96±0.1
1	0.99±0.1	1.04±0.1	0.98±0.1
2	1.08±0.1	0.89±0.1	1.08±0.1
5	1.03±0.2	1.28±0.2	2.04±0.2

Table S3. Fold induced RFP vs. MS concentration (mM) for each variant. Fold induced RFP indicates the normalized fluorescence value in the presence of the inducer divided by the normalized fluorescence value in the absence of the inducer. Data points are the average of 3 values.

		Fold induced RFP of the variants (N=3, ±SD)									
**MS concentration (mM)**	WT	Var1	Var5	Var6	Var7	Var8	Var9	Var10	Var2	Var3	Var4
0	1	1	1	1	1	1	1	1	1	1	1
0.1	1.0±0.1	1.2±0.2	2.2±0.4	1.8±0.2	2.2±0.6	2.7±0.1	2.4±0.2	1.9±0.2	1.2±0.2	0.9±0.1	1.1±0.1
0.5	1.1±0.1	4.7±0.8	9.8±2	9.4±0.7	11.3±1	7.5±0.3	8.7±0.4	10.2±1	4.7±0.8	1.3±0.2	3.0±0.2
1	1.0±0.1	9.3±1	15.1±2	15.2±1	17.3±2	9.6±1	11.2±1	15±2	9.3±1	1.8±0.2	6.4±1

2	1.6±0.1	13.9±1	19±2	19.9±1	22.8±3	11.7±1	13.3±1	18.0±3	13.9±1	3.3±0.2	10.9±1
5	2.5±0.2	17.4±1	23.1±3	22.9±1	25.0±3	12.8±1	14.4±1	20.3±2	17.4±1	5.1±0.2	14.1±2

Table S4. The Glide docking result parameters.

	**Glide docking result parameters (kcal/mol)**									
**Receptor–ligand**	**docking_score**	**glide_gscore**	**glide_lipo**	**glide_hbond**	**glide_rewards**	**glide_evdw**	**glide_ecoul**	**glide_erotb**	**glide_esite**	**glide_emodel**
**WT-ItcR-IA**	-5.32	-5.32	-0.80	-0.72	-2.37	-3.96	-19.37	1.68	-0.004	-44.98
**WT-ItcR-MS-S**	-4.01	-4.01	-0.53	-0.67	-2.96	-17.6	-9.14	2.39	0.000	-31.31
**Var7-IA**	-4.90	-4.90	-0.81	-0.36	-3.00	-9.12	-12.96	1.68	0.000	-36.16
**Var7-MS-R**	-5.47	-5.47	-0.68	-0.46	-2.91	-3.36	-13.75	0.82	0.000	-29.65
**Var7-MS-S**	-5.86	-5.86	-0.82	-0.53	-2.59	-8.54	-15.36	0.82	-0.002	-38.05

References

1. Sun, P., et al., *A genetically encoded fluorescent biosensor for detecting itaconate with subcellular resolution in living macrophages.* Nature Communications, 2022. **13**(1).
2. Shaner, N.C., et al., *Improved monomeric red, orange and yellow fluorescent proteins derived from Discosoma sp. red fluorescent protein.* Nature Biotechnology, 2004. **22**(12): p. 1567-1572.
